# Identification of Thyroid Genes Whose Expression Is Altered by Neonatal Irradiation in Rats

**DOI:** 10.3390/ijms26051874

**Published:** 2025-02-21

**Authors:** Nariaki Fujimoto, Mutsumi Matsuu-Matsuyama, Masahiro Nakashima

**Affiliations:** 1 Research Institute for Radiation Biology and Medicine, Hiroshima University, Hiroshima 734-8553, Japan; 2 Atomic Bomb Disease Institute, Nagasaki University, Nagasaki 852-8523, Japan; mutsumi@nagasaki-u.ac.jp (M.M.-M.); moemoe@nagasaki-u.ac.jp (M.N.)

**Keywords:** neonatal X-irradiation, childhood radiation, thyroid carcinogenesis

## Abstract

Childhood radiation is a risk factor for thyroid cancer that became well known after the Chernobyl nuclear plant accident. Although these human cases have been extensively studied, the mechanisms underlying childhood susceptibility to radiation-induced thyroid cancer have yet to be explained. Our previous study showed that neonatal X-irradiation resulted in long-term alterations in the mRNA expression of thyroid cancer-related marker genes, which may be a critical mechanism for understanding the higher radiation sensitivity in young patients. In this study, RNA sequencing (RNA-Seq)-based gene expression analysis was employed to identify thyroid genes whose mRNA expression was changed by neonatal irradiation. Male Wistar rats aged 1 week and 4 months were subjected to cervical X-irradiation at 4 Gy. After 8 weeks, total RNA was extracted from the thyroid and subjected to RNA-Seq analysis to identify differentially expressed genes following irradiation. We identified five upregulated genes (i.e., *Adm2*, *Vnn1*, *Snph*, *Gria3*, and *Cpa4*) and one downregulated gene (i.e., *Crtac1*) explicitly altered by neonatal radiation exposure. Western blotting confirmed the corresponding changes in CPA4 and CRTAC1 expression. The gene expressions identified were also altered in thyroid tumors induced by an iodine-deficient diet. These long-term changes in thyroid gene expression caused by neonatal irradiation may be involved in the increased risk of thyroid carcinogenesis.

## 1. Introduction

Radiation exposure at a young age is a risk factor for thyroid carcinogenesis [1,2]. Early studies of infants treated with X-rays and a cohort study of Japanese atomic bomb survivors have suggested an association between thyroid cancer and childhood radiation [3,4]. The risk was acknowledged again following the Chernobyl nuclear plant accident, in which papillary thyroid cancer cases rapidly increased among the exposed children population [5,6]. However, the mechanisms underlying childhood susceptibility to radiation-induced thyroid cancer have not yet been clarified. A higher rate of mitosis of thyroid follicular cells in the neonatal period has been implicated in the high prevalence of thyroid cancer [7,8]. Although the activation of *RET* and *BRAF* by either point mutations or chromosomal rearrangements was frequently found in post-Chernobyl thyroid cancer cases, they are also common in sporadic papillary thyroid cancer [9,10]. In the laboratory, neonatal rats exposed to cervical X-irradiation were found to develop thyroid cancer with a significantly higher incidence than adult animals [11]. We investigated the thyroid of 1-week-old Wistar rats cervically X-irradiated compared with 8-week-old rats [12,13]. We found that single neonatal irradiation induced long-term changes in the thyroidal mRNA expression of thyroid cancer-related genes, including *Mct8*, *Lat2/4*, and *Lgals*. Interestingly, these gene expression alterations were also found in thyroid tumors induced by an iodine-deficient diet (IDD).

We postulated that genes whose expression is preferentially altered by neonatal thyroid irradiation are related to childhood susceptibility to radiation-induced thyroid cancer. To identify these genes, 1-week-old and 4-month-old Wistar rats were subjected to sham exposure or 4 Gy of cervical X-irradiation. After 8 weeks, total RNA was extracted from the thyroid, and global gene expression analysis using RNA sequencing (RNA Seq) was performed to determine differentially expressed genes (DEGs).

## 2. Results

### 2.1. Body and Thyroid Weights and Serum TT3, TT4, and TSH Levels

Table 1 presents body and thyroid weights at necropsy. After sham or X-irradiation, body weights steadily increased in all groups. No significant differences were observed between the sham- and X-irradiated groups in either age. Supplementary Table S1 presents serum total T3, total T4, and TSH levels at necropsy. Either hormone level did not change by cervical irradiation at 4 Gy in either age group.

### 2.2. Identification of DEGs 8 Weeks After Irradiation

**Figure 1:**
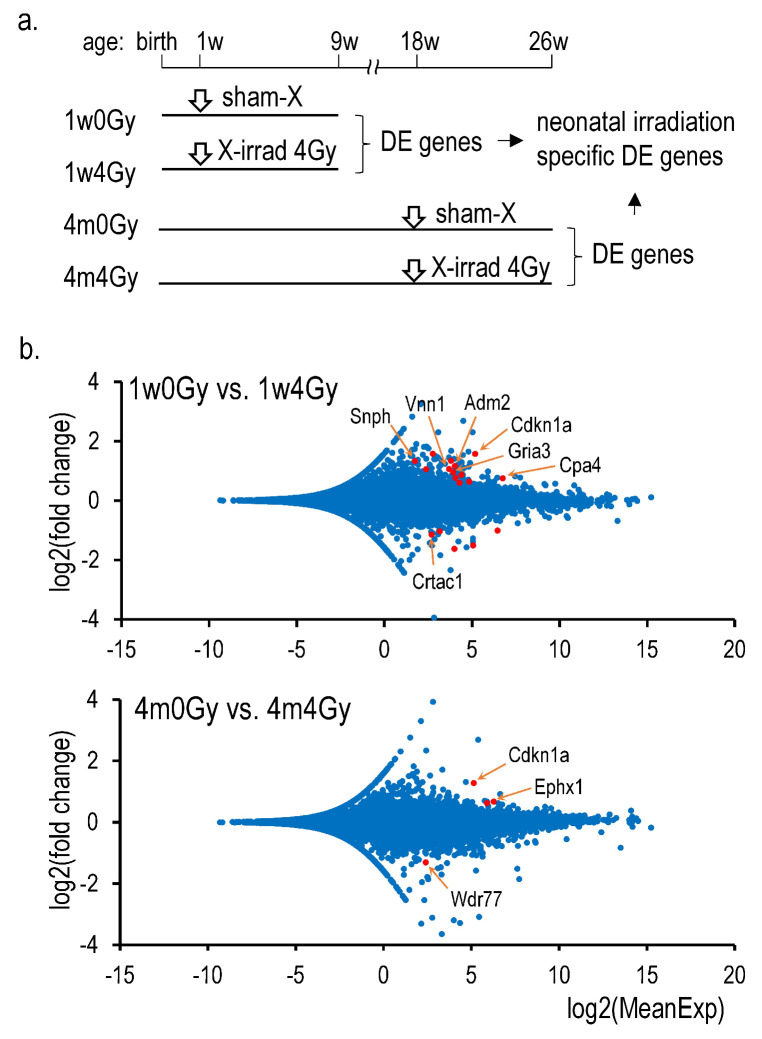
RNA-Seq analysis in the X-irradiated rat thyroid. (**a**): Schedule of the experiment, (**b**): MA plots consisting of 18,982 dots (above) and 18,989 dots (below) representing all mapped genes. Red dots indicate differentially expressed genes identified by the DEseq2 analysis, as shown in Supplementary Table S2 (Only Q-RT-PCR-validated gene names were shown).

Eight samples of total RNAs from the thyroid were sequenced, each two samples from 1w0Gy, 1w4Gy, 4m0G, and 4m4Gy. The RINs of these samples ranged from 8.7 to 9.1. The paired reads were between 20 M and 24 M, and 96.9% were mapped in all samples. DEGs were determined using the DEseq2 (Wald/parametric) of the ‘RaNA-Seq’ platform [14] between 1w0Gy and 1w4Gy or between 4m0Gy and 4m4Gy. Genes with >1.5- and <0.5-fold changes were selected. Figure 1 presents the results as MA plots. By neonatal irradiation, thirteen upregulated and five downregulated genes were identified, whereas only three upregulated and one downregulated gene was identified in the rat thyroid samples irradiated at 4 months of age. The changes in the mRNA expression levels of these genes were validated using Q-RT-PCR (Table S2). Table 2 presents the validated genes. Because the increase in Cdkn1a expression was not neonatal irradiation-specific, it is concluded that *Snph*, *adm2*, *Vnn1*, *Gria3*, *Cpa4*, and *Crtac1* are genes whose expression is altered explicitly by neonatal cervical X-irradiation. The RNA-Seq data were deposited in the Gene Expression Omnibus database under the accession number GSE284022.

### 2.3. Expression of Genes in the Thyroid Exposed to X-Rays at Different Ages

The thyroidal mRNA expression of the identified genes was determined in rats exposed to X-rays at 4 and 8 weeks of age and at 4 months of age: 4w0Gy, 4w4Gy, 8w0Gy, 8w4Gy, 4m0Gy, and 4m4Gy (Table 3). In rats exposed to 4 Gy of X-rays at 4 weeks of age, only the *Snph* mRNA level was significantly increased compared with that in the sham exposure group, whereas the expression of the other genes did not change. In the 8w4Gy or 4m4Gy groups, the expression of the identified genes was not changed after irradiation.

### 2.4. CPA4 and CRATC1 Protein Expression in the X-Irradiated Thyroid

**Figure 2:**
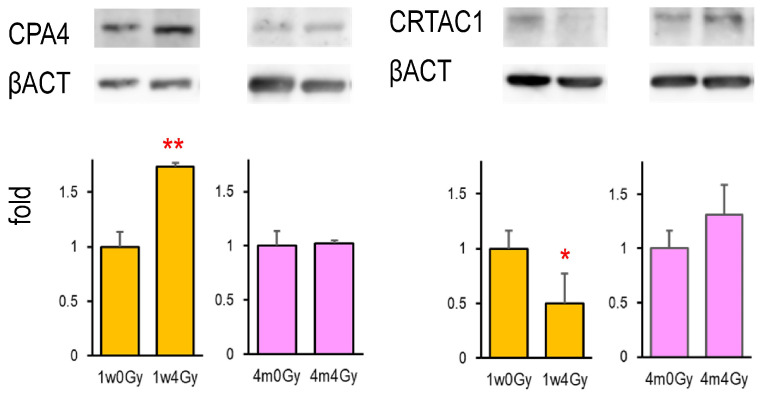
CPA4 and CRTAC1 protein expressions in the thyroid in rats cervically X-irradiated at 1 week and 4 months old. Lysates extracted from the thyroid were examined by Western blot, *, ** indicate significant differences from the control (0 Gy) at *p* < 0.05 or *p* < 0.01, respectively. *n* = 5 (1w0Gy), *n* = 4 (1w4Gy), *n* = 4 (4m0Gy), and *n* = 4 (4m4Gy).

Among the six identified genes, only *Cpa4* and *Crtac1* gene products were detectable using Western blotting. Figure 2 shows that CPA4 protein expression was elevated by neonatal X-irradiation, whereas CRTAC1 expression was decreased. In contrast, in the rat thyroid exposed to X-rays at 4 months of age, the expression of either protein was not significantly changed after irradiation. The original Western blot images were presented in Figures S1 and S2.

### 2.5. Radiation Dose-Dependent Changes in mRNA Expression of Identified Genes

**Figure 3:**
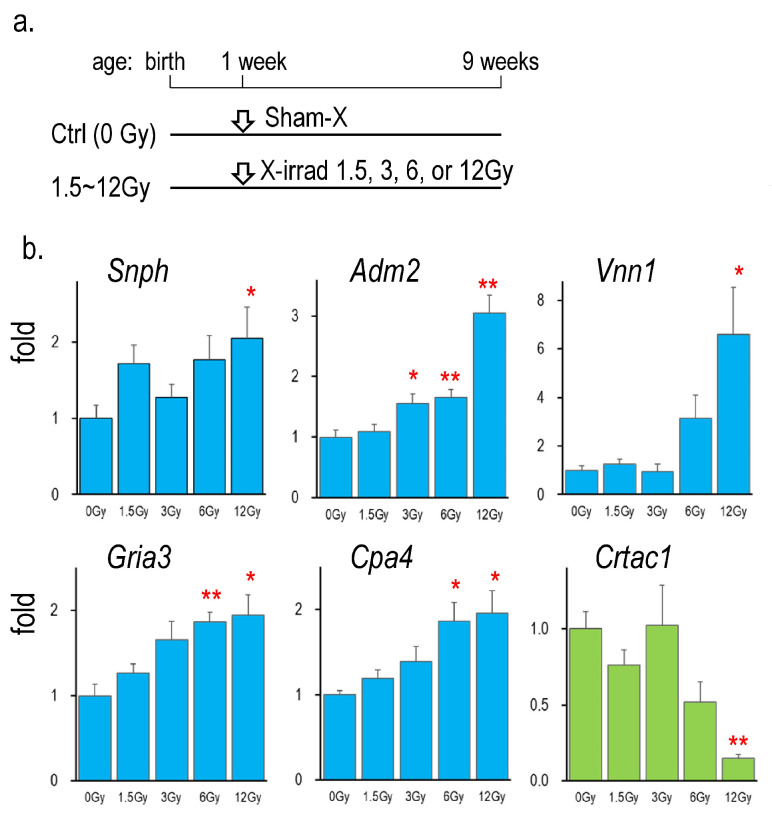
Gene expressions in the thyroid of rats neonatally exposed to various doses of X-rays. (**a**): Schedule of the experiment [13], (**b**): Thyroidal mRNA levels of rats X-irradiated at 0, 1.5, 3, 6, and 12 Gy, *, ** indicate significant differences from the control (0 Gy) at *p* < 0.05, or *p* < 0.01, respectively. *n* = 6, each group.

Figure 3 shows the mRNA expression of *Snph*, *adm2*, *Vnn1*, *Gria3*, *Cpa4*, and *Crtac1* in the thyroid of rats neonatally exposed to different doses of X-rays ranging from 1.5 to 12 Gy. All gene expressions were changed by neonatal irradiation in a radiation dose-dependent manner.

### 2.6. Changes in the Expression of Identified Genes in Thyroid Tumors Induced by IDD Feeding

**Figure 4:**
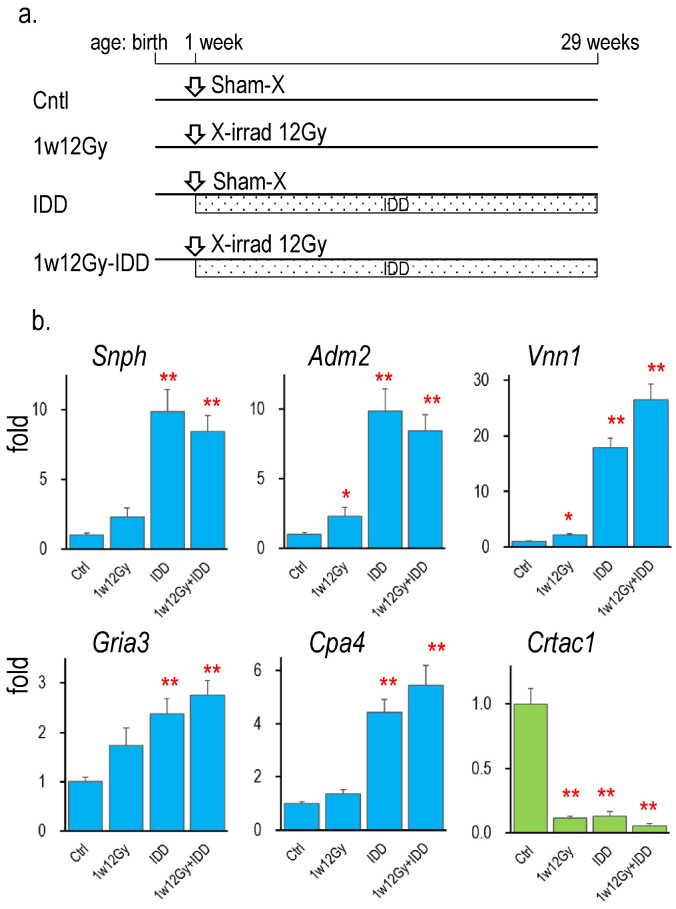
Gene expressions in the thyroid of rats neonatally X-irradiated (1w12Gy), fed with IDD (IDD), or both (1w12Gy-IDD). (**a**): Schedule of the experiment [13], (**b**): Changes in the identified gene’s mRNA levels in the thyroid relative to the control, *, ** indicate significant differences from the control (0 Gy) at *p* < 0.05 or *p* < 0.01, respectively. *n* = 4 (Cntl), *n* = 3 (1w12Gy), *n* = 7 (IDD), and *n* = 8 (1w12Gy-IDD).

The expression of identified genes was further examined in the thyroid tumor samples of our previously reported study [13], in which neonatal rats were cervically exposed to either sham or 12 Gy of X-rays and were fed IDD for 28 weeks. Figure 4 presents gene expression relative to the control (sham irradiation/standard diet). At 28 weeks after neonatal X-irradiation, the increase in *Adm2* and *Vnn1* mRNA levels and the decrease in *Crtac1* mRNA expression continued. In thyroid tumors induced by IDD, the expression of every identified gene was significantly changed. The increase in *Vnn1* and the decrease in *Crtac1* were further enhanced by the combination of X-irradiation and IDD (1w12Gy + IDD group). In contrast, the expression of the other genes was similarly higher in the IDD and 1w12Gy + IDD groups than in the 1w12Gy group.

## 3. Discussion

Although the association between thyroid cancer risk and childhood radiation exposure has been well established, the underlying mechanisms remain unclear. Our previous investigation suggested that neonatal radiation exposure alters thyroidal gene expression in the long term and is an essential clue to understanding the susceptibility of young patients to thyroid cancer. In this study, global gene expression analysis was performed to identify genes whose expression was altered explicitly by neonatal radiation exposure. DEGs were detected in the thyroid 8 weeks after neonatal X-irradiation. In contrast, only a few DEGs were identified in the adult thyroid 8 weeks after X-irradiation. This study successfully demonstrated that neonatal radiation exposure preferentially induces long-term gene expression changes.

In a previous study, we investigated rats cervically exposed to 12 Gy of X-rays during the neonatal period and found distinctive effects on thyroid histology [12]. At 8 weeks after neonatal irradiation, thyroid follicle size was significantly reduced with thickening and fusion of epithelial cells, whereas no histological changes were observed in the case of adult exposure to X-rays. Furthermore, 12-Gy neonatal irradiation affected thyroid functions because Tg protein expression was reduced, although serum TSH levels changed only temporarily. These findings with 12-Gy exposure may coincide with the epidemiological data of a Ukrainian cohort, showing an increase in hypothyroidism cases among those who received high thyroid doses of 5–27-Gy radiation during childhood [15]. However, the estimated average thyroid doses in child evacuees of the Chernobyl accident ranged from 1.0 to 3.1, depending on the region [16]. Veiga et al. comprehensively reviewed thyroid cancer after childhood medical radiation and the atomic bomb, finding that relative risks increased with 2–4 Gy [17]. Therefore, this study used a more realistic thyroid dose—4 Gy of cervical X-irradiation—for the global gene expression analysis. A Wistar rat study demonstrated that thyroid cancer developed at an incidence of 33% 16 months after irradiation with 4 Gy at 7 weeks of age [18]. Other studies have also reported that thyroid cancer could be induced by 3 Gy of X-irradiation combined with the administration of a thyroid peroxidase inhibitor [11,19]. In this study, no differences in body and thyroid weights or serum TT3, TT4, or TSH levels were observed between 1w0Gy and 1w4Gy, or between 4m0Gy and 4m4Gy. However, exposure to 12-Gy neonatal radiation evidently affected the growth rate, as we previously reported [12].

The RNA-Seq analysis, followed by validation using Q-RT-PCR, successfully identified DEGs between 1w0Gy and 1w4Gy or between 4m0Gy and 4m4Gy (Table 2). Comparing the results of irradiation at the neonatal period and at 4 months of age, one gene, *Cdkn1a*, was common. Therefore, the other six genes in Table 2—*Snph*, *Adm2*, *Vnn1*, *Gria3*, *CPa4*, and *Crtac1*—were recognized as genes whose expression was specifically altered by neonatal irradiation. They were further examined in the thyroid of rats irradiated at older ages (Table 3), which showed that these alterations in mRNA expression were not observed in those irradiated at 4 weeks of age or older, except for *Snph*, which was inducible at 4 weeks of age. The radiation dose-responsive changes in the identified gene expression were confirmed in independent animal experiments (Figure 4), demonstrating the reproducibility of the results. It is worth noting that these gene expression changes were still evident even 28 weeks after neonatal irradiation, as shown in Figure 4 (control versus 1w12Gy). Our Western blot analysis detected only protein products of *Cpa4* and *Crtac1* genes out of the identified six. Since the mRNA expression levels of these genes were similar, except for *Cpa4* being high, the post-transcriptional mechanisms may differ. Although Wistar rats have been widely used for thyroid cancer studies, the incidence and morphology of thyroid tumors vary among different rat strains [20]. Future studies need to examine thyroidal gene expressions in the other rat strains to understand their attributions to radiation exposure.

*Snph* encodes synphilin, which was originally identified as a protein that controls mitochondrial movement in neurons [21]. Although synphilin is primarily expressed in the brain, it may be related to cancer cell proliferation and metastasis, such as prostate cancer [22]. Adrenomedullin 2 (ADM2) is a member of the calcitonin superfamily and is expressed in various organs throughout the body [23]. It is a peptide ligand that binds to both calcitonin receptor-like receptors and calcitonin receptors to exhibit various effects, including vasodilation and angiogenic effects. In rats with thyroid hyperplasia induced by IDD feeding, the expression of AMD2 was found to be markedly increased [24]. They further showed that TSH directly upregulates the secretion of ADM2 in the thyroid cell line FRTL-5. A recent investigation of a mouse thyroid cancer model with thyrocyte-specific activation of BRAF^V600E^ revealed that ADM2 was upregulated in thyroid tumors [25]. This study showed that the gene expression of *Adm2* was increased only by neonatal irradiation and continued to be upregulated even at 6 months after exposure, suggesting the critical role of Adm2 in the development of thyroid cancer by childhood irradiation. Vanin 1 (VNN1) is a ubiquitously expressed enzyme that degrades pantetheine in cysteamine and pantothenic acid [26]. Physiologically, it contributes to stress responses. Although studies have suggested that the expression of *Vnn1* serves as a biomarker for tumor prognosis, the roles of VNN1 in carcinogenesis, if any, are unknown [27]. Glutamine receptor A3 (GRIA3) regulates a broad spectrum of brain and nervous events [28]. A recent bioinformatics study found that Gria3 expression is a useful prediction marker of radiation dose in the kidney [29]. Carboxypeptidase A4 (CPA4) is a Zn-containing metallocarboxypeptidase that catalyzes carboxy-terminal amino acids. It is a critical regulator of inflammation and is upregulated in gastric and breast cancers [30,31]. The expression of *Cpa4* is higher in human thyroid cancer than in normal and benign tissues and is associated with poor prognosis and dedifferentiation [32]. *Cpa4* expression is also useful for diagnosing breast cancer [30]. Cartilage acidic protein 1 (CRTAC1), an extracellular matrix protein, could be a tumor suppressor. In urothelial carcinoma, low CRTAC1 expression is significantly associated with high tumor stage, vascular invasion, and nodule metastasis [33]. The mRNA expression of CRTAC1 was lower in lung adenocarcinoma than in normal tissues, and reduced expression levels were associated with poor prognosis [34]. A cell culture study demonstrated that CRTAC1 overexpression inhibits cell proliferation, migration, invasion, and epithelial–mesenchymal transition process of bladder cells by downregulating the expression of the YY1 protein [35]. Decreased expression following neonatal irradiation may contribute to the progression of thyroid cancer. Although the possible involvement of the identified genes in thyroid carcinogenesis is suggested above, the significance of our findings needs to be vilified in thyroid lesions of human specimens in the future. In addition to the gene’s cellular function, the systemic effects of the expression changes will need to be investigated.

In rats, thyroid tumor development is strongly associated with serum levels of TSH, which acts mainly as a tumor promoter. We previously examined a thyroid tumor model in rats fed with IDD, which exhibited continuously elevated serum TSH levels through the hypothalamus–pituitary feedback. The incidence of thyroid adenoma reached 75% in 28 weeks after the combination of neonatal X-irradiation and IDD feeding, whereas hyperplasia was observed only in the IDD alone group [13]. Surprisingly, the expression of all identified genes was significantly changed in thyroid hyperplasia induced by IDD alone, suggesting that neonatal X-irradiation altered the expression of genes commonly induced by the tumor promotion of TSH. The expression of *Vnn1* and *Crtac1* was further enhanced by neonatal irradiation, which may have contributed to the transition from hyperplasia to adenoma observed in the 1w12Gy group. Despite the gene expression similarity, serum TSH was not involved in the present study (Supplementary Table S1). Our previous study of rats exposed to 8 Gy of X-rays did not show any changes in serum TSH levels during the 18 months of study [36].

*In vivo* investigations have shown that exposure to ionizing radiation can lead to long-term gene expression changes [37,38]. A recent comprehensive transcriptomic analysis of mice exposed to 1 Gy of X-rays demonstrated that the expression of many genes was altered 10 weeks post-exposure, although the expression profiles differed depending on the mouse strain and target organ [39]. Epigenomic mechanisms likely explain these long-term gene expression changes [40,41]. Larsson et al. investigated the transcriptomic and proteomic changes in the thyroid of rats internally exposed to ^131^I and successfully identified DEGs at 9 months post-exposure [42]. These alterations in gene expression may be related to the disruption of thyroid functions and possible thyroid cancer development. We postulated that alterations in gene expression caused by radiation exposure are significantly different during the neonatal period from those during the adult period, which may be related to neonatal susceptibility to thyroid carcinogenesis. In this study, we successfully identified genes whose expression was altered by only “neonatal irradiation” in the long term.

## 4. Materials and Methods

### 4.1. Animals

Wistar rats were purchased from Jackson Laboratory Japan (Yokohama, Japan) and used after an acclimatization period of 1 week. The animals were maintained with free access to a basal diet (MF, Oriental Yeast Co., Tokyo, Japan) and tap water at room temperature of 23.0 ± 2.0 °C, relative humidity of 50.0 ± 10.0%, and a 12 h light cycle. To obtain 5- or 6-day-old rats (designated as 1-week-old), 3-month-old pregnant Wistar rats (16–18 days of gestation) were purchased, and male pups were selected after delivery. Four-week-old, eight-week-old, and four-month-old male Wistar rats were used as their counterparts. Animals were housed two or three in each cage. Rats in different age groups were randomly divided into two groups. They were exposed to sham or 4 Gy of cervical X-irradiation, designated as 1w0Gy (*n* = 7), 1w4Gy (*n* = 7), 4w0Gy (*n* = 5), 4w4Gy (*n* = 5), 8w0Gy (*n* = 5), 8w4Gy (*n* = 5), 4m0Gy (*n* = 7), and 4m4Gy (*n* = 7), respectively. After approximately 8 weeks, the animals were euthanized by whole blood removal from an abdominal artery under anesthesia with isoflurane inhalation. Thyroid tissues were dissected and stored in an RNA Save solution (Biological Industries, Cromwell, CT, USA) for RNA extraction. The experiments were repeated for 1w0Gy (*n* = 5), 1w4Gy (*n* = 4), 4m0Gy (*n* = 4), and 4m4Gy (*n* = 4) to obtain frozen thyroid tissue samples stored at −80 °C for protein extraction. The total number of animals was 65. The animal experiments were approved by the Animal Experiment Committee of Hiroshima University (document # A21-111, 21 September 2021). This study was conducted according to the Guide for the Care and Use of Laboratory Animals at Hiroshima University. The study was conducted according to the Animal Research: Reporting of In Vivo Experiments (ARRIVE) guidelines (Supplementary File S1).

### 4.2. Cervical X-Irradiation

The details were described previously [12]. Lead plates of 2 mm in thickness were used to cover the animals with a gap for the neck region. Irradiation was performed using the X-ray irradiator MBR-1520R-3 (Hitachi Medical Co., Tokyo, Japan) at a dose rate of 0.9 Gy/min.

### 4.3. Serum T3, T4, and Thyroid-Stimulating Hormone (TSH)

Total T3 and T4 were determined using enzyme-linked immunosorbent assay kits from Alpco Diagnostics (Salem, NH, USA). Serum TSH levels were measured using a radioimmunoassay kit from the National Hormone and Peptide Program (NIDDK, NIH, Torrance, CA, USA). TSH was radiolabeled using the lactoperoxidase method with Na^125^I (PerkinElmer, Shelton, CT, USA). Donkey antirabbit serum which was the second antibody, was purchased from Immundiagnostik AG (Bensheim, Germany).

### 4.4. RNA-Seq Analysis

Total RNA was isolated from thyroid tissue using Isogen II (Nippon Gene Co., Tokyo, Japan) according to the manufacturer’s instructions. The 2100 Bioanalyzer (Agilent Technologies, Santa Clara, CA, USA) was used to assess the quality of the prepared total RNA to determine the RIN in each sample from different rats. Two total thyroidal RNA samples with higher RINs were selected from seven samples, each group of 1w0Gy, 1w4Gy, 4m0Gy, or 4m4Gy. They were subjected to RNA Seq, which was performed by Bioengineering Lab. Co., Ltd. (Sagamihara, Japan). In brief, cDNA libraries were prepared using the MGIEasy RNA Directional Library Prep Set (MGI Tech Co., Ltd., Shenzhen, China) and circularized using the MGIEasy Circularization Kit (MGI Tech Co.). DNA nanoballs (DNBs) were produced using a DNBSEQ-G400RS High-Throughput Sequencing Kit (MGI Tech Co.) and sequenced using 100 bp paired-end analysis of 20 million reads by DNBSEQ-G400 (MGI Tech Co.). Genomic mapping was performed using hisat2 (ver. 2.2.1). Reads were counted using featureCounts (ver. 2.0.0) and normalized as transcripts per million. Comparisons between 1w0Gy and 1w4Gy and between 4m0Gy and 4m4Gy were performed using DEseq2 with the Wald test and fit type of “parametric” (https://ranaseq.eu/, accessed on 10 January 2024).

### 4.5. Quantitative RT-PCR

cDNA was synthesized by incubating 3-µg of total RNA with 100 U of ReverTra Ace reverse transcriptase (Toyobo Co., Osaka, Japan) in a mixture of 20 pmol of random hexamers pdN6 and 5-pmol oligo-dT(15) primers (Takara Bio Inc., Kusatsu, Japan). A quantitative PCR instrument, StepOnePlus (Applied Biosystems/Life Technologies Co., Carlsbad, CA, USA), was used to measure cDNA levels with THUNDERBIRD Next SYBR q-PCR Mix (Toyobo Co.). Supplementary Table S3 presents the sequences of the specific primer sets. The DNA sequences of the PCR products were confirmed by Fasmac Co., Ltd. (Atsugi, Japan). The PCR conditions included an initial denaturation for 2 min, followed by 40 cycles of 5 s incubation at 95 °C and 35 s incubation at 60 °C. The measured mRNA levels were normalized against the levels of β-actin mRNA [13].

### 4.6. Western Blotting

Thyroid tissues were homogenized in RIPA lysis buffer containing phosphatase inhibitors (Santa Cruz Biotechnology Inc., Santa Cruz, CA, USA). Two g of each lysate were applied to 12.5% SDS-PAGE and transferred to a 0.2-µm ClearTrans SP PVDF membrane (Fujifilm Wako Pure Chemical Co., Osaka, Japan). After blocking with Blocking One (Nacalai Tesque Inc., Kyoto, Japan), the membranes were incubated with anti-CPA4 (NBP2-92372, 1:1000, Novus biologicals, Centennial, CO, USA), anti-CRTAC1 (30251-1-AP, 1:1000, Proteintech Group, Inc., Rosemont, IL, USA), or anti-βActin (3H12, 1:1000, MBL Co., Nagoya, Japan). They were then washed and incubated with peroxidase-conjugated antirabbit IgG (1:4000, MBL Co.). The protein bands were visualized using Chemi-Lumi reagents (Nacalai Tesque Inc.) and images were captured using a CCD camera system, ImageQuant LAS 4000mini (GE Healthcare Ltd., Buckinghamshire, UK). The band intensities were quantified using ImageJ version 1.53a (http://imagej.nih.gov, accessed on 7 June 2024).

### 4.7. Statistical Analysis

All values are expressed as means ± standard errors. Student’s *t*-test was used to compare the two groups with Excel 2019 software. Dunnett’s test was applied for multiple comparisons among the group with the R’s package ‘SimComp’ version 3.3 (http://cran.r-project.org, accessed on 15 October 2024).

## 5. Conclusions

Irradiation causes long-term gene expression changes in the thyroid, particularly during the neonatal period. The thyroid glands of rats 8 weeks after irradiation were subjected to comprehensive gene expression analysis. By comparing the results between neonate and adult irradiation, we successfully identified long-term changes in gene expression, particularly those after exposure to neonatal irradiation. Because these changes were common with those observed in thyroid tumors, the identified genes and their altered expression may be factors associated with childhood susceptibility to irradiation-induced thyroid cancer.

## Figures and Tables

**Table 1:** Body and thyroid weights of rats.

Group	Treatment	Initial Body Weights (g)	Final Body Weights (g)	Thyroid Weight (mg)	Thyroid Weight (mg/kg b.w.)	
1 week old						
1w0Gy	Sham-X	15 ± 0.3	364 ± 9.0	18 ± 0.9	50 ± 2.7	RNA-seq
1w4Gy	4 Gy	15 ± 0.3	349 ± 7.9	15 ± 1.0	42 ± 3.4	
4 weeks old						
4w0Gy	Sham-X	52 ± 0.9	456 ± 21.5	20 ± 2.2	42 ± 2.7	
4w4Gy	4 Gy	53 ± 1.8	447 ± 8.7	19 ± 1.0	42 ± 2.2	
8 weeks old						
8w0Gy	Sham-X	301 ± 3.4	522 ± 11.0	22 ± 3.7	49 ± 6.0	
8w4Gy	4 Gy	305 ± 2.4	545 ± 17.9	28 ± 3.0	51 ± 4.9	
4 months old						
4m0Gy	Sham-X	575 ± 17.7	659 ± 17.5	29 ± 2.0	43 ± 2.8	RNA-seq
4m4Gy	4 Gy	576 ± 13.8	647 ± 19.7	29 ± 1.7	45 ± 1.8	

The numbers of rats were 7 rats/group (1 w and 4 m) and 5 rats/group (4 w and 8 w).

**Table 2:** (**a**): Genes whose thyroidal expressions were altered by cervical irradiation. (**b**): Identified genes whose thyroidal expressions were altered by cervical irradiation.

Gene	RNA-Seq		Q-PCR	
	Fold	Padj	Fold Change	
(a) at 1 week old				
Up-regulated				
*Cdkn1a*	3.08	0.000	2.3 ± 0.14 **	Cyclin-dependent kinase inhib 1A
*Snph*	2.43	0.001	1.9 ± 0.24 **	Syntaphilin
*Adm2*	2.37	0.019	1.9 ± 0.23 **	Adrenomedullin 2
*Vnn1*	2.22	0.011	1.8 ± 0.24 *	Vanin 1
*Gria3*	2.03	0.000	1.5 ± 0.06 **	Glutamine receptor A3
*Cpa4*	1.69	0.013	1.4 ± 0.09 **	Carboxypeptidase A4
Down-regulated				
*Crtac1*	0.39	0.009	0.44 ± 0.03 *	Cartilage acidic protein 1
(b) at 4 months old				
Up-regulated				
*Cdkn1a*	2.49	0.000	2.3 ± 0.21 **	Cyclin-dependent kinase inhib 1A
*Ephx1*	1.60	0.000	1.4 ± 0.08 **	Epoxide hydrolase 1
Down-regulated				
*Wdr77*	0.33	0.033	0.83 ± 0.03 *	WD repeat domain 77

*, ** indicate statistical differences from each control (0 Gy) at *p* < 0.05 (*) or *p* < 0.01 (**); *n* = 2, each, in RNA-Seq, *n* = 7, each, in Q-PCR analysis.

**Table 3:** Relative thyroidal mRNA expressions in X-irradiated rats at different ages.

Gene	1w4Gy	4wGy	8w4Gy	4m4Gy
Up-regulated				
*Snph*	1.9 ± 0.24 **	1.4 ± 0.12 *	1.1 ± 0.11	1.2 ± 0.19
*Adm2*	1.9 ± 0.23 **	0.93 ± 0.13	0.92 ± 0.26	0.84 ± 0.16
*Vnn1*	1.8 ± 0.24 *	1.1 ± 0.18	0.83 ± 0.20	0.99 ± 0.17
*Gria3*	1.5 ± 0.06 **	1.0 ± 0.12	1.0 ± 0.09	0.79 ± 0.09
*Cpa4*	1.4 ± 0.09 **	0.74 ± 0.13	0.92 ± 0.16	0.85 ± 0.1
Down-regulated				
*Crtac1*	0.44 ± 0.03 *	1.07 ± 0.12	0.68 ± 0.13	0.94 ± 0.14

*, ** indicate statistical differences from each control (0 Gy) at *p* < 0.05 (*) or *p* < 0.01 (**). *n* = 7, each, in 1 w and 4 m groups, and *n* = 5, each, in 4 w and 8 w groups.

## Data Availability

The RNA-Seq data were deposited in the Gene Expression Omnibus database under the accession number GSE284022. All data are presented in the article and the Supplementary Materials.

## Supplementary Materials

The following supporting information can be downloaded at: https://www.mdpi.com/article/10.3390/ijms26051874/s1.

## References

[1] Drozdovitch V. (2021). Radiation Exposure to the Thyroid After the Chernobyl Accident. Front. Endocrinol..

[2] Reiners C. (2009). Radioactivity and thyroid cancer. Hormones.

[3] Furukawa K., Preston D., Funamoto S., Yonehara S., Ito M., Tokuoka S., Sugiyama H., Soda M., Ozasa K., Mabuchi K. (2012). Long-term trend of thyroid cancer risk among Japanese atomic-bomb survivors: 60 years after exposure. Int. J. Cancer.

[4] Raventos A., Winship T. (1964). The Latent Interval for Thyroid Cancer Following Irradiation. Radiology.

[5] Brenner A.V., Tronko M.D., Hatch M., Bogdanova T.I., Oliynik V.A., Lubin J.H., Zablotska L.B., Tereschenko V.P., McConnell R.J., Zamotaeva G.A. (2011). I-131 Dose Response for Incident Thyroid Cancers in Ukraine Related to the Chornobyl Accident. Environ. Health Perspect..

[6] Zablotska L.B., Bogdanova T.I., Ron E., Epstein O.V., Robbins J., Likhtarev I.A., Hatch M., Markov V.V., Bouville A.C., Olijnyk V.A. (2007). A Cohort Study of Thyroid Cancer and Other Thyroid Diseases after the Chornobyl Accident: Dose-Response Analysis of Thyroid Follicular Adenomas Detected during First Screening in Ukraine (1998–2000). Am. J. Epidemiol..

[7] Sikov M.R. (1969). Effect of Age on the Iodine-131 Metabolism and the Radiation Sensitivity of the Rat Thyroid. Radiat. Res..

[8] Williams D. (2015). Thyroid Growth and Cancer. Eur. Thyroid. J..

[9] Abdullah M.I., Junit S.M., Ng K.L., Jayapalan J.J., Karikalan B., Hashim O.H. (2019). Papillary Thyroid Cancer: Genetic Alterations and Molecular Biomarker Investigations. Int. J. Med. Sci..

[10] Thomas G. (2018). RADIATION AND THYROID CANCER—AN OVERVIEW. Radiat. Prot. Dosim..

[11] Christov K. (1978). Radiation-Induced Thyroid Tumors in Infant Rats. Radiat. Res..

[12] Fujimoto N., Matsuu-Matsuyama M., Nakashima M. (2020). Morphological and functional changes in neonatally X-irradiated thyroid gland in rats. Endocr. J..

[13] Fujimoto N., Matsuu-Matsuyama M., Nakashima M. (2021). Single neonatal irradiation induces long-term gene expression changes in the thyroid gland, which may be involved in the tumorigenesis. Sci. Rep..

[14] Prieto C., Barrios D. (2020). RaNA-Seq: Interactive RNA-Seq analysis from FASTQ files to functional analysis. Bioinformatics.

[15] Ostroumova E., Rozhko A., Hatch M., Furukawa K., Polyanskaya O., McConnell R.J., Nadyrov E., Petrenko S., Romanov G., Yauseyenka V. (2013). Measures of Thyroid Function among Belarusian Children and Adolescents Exposed to Iodine-131 from the Accident at the Chernobyl Nuclear Plant. Environ. Health Perspect..

[16] Cardis E., Howe G., Ron E., Bebeshko V., Bogdanova T., Bouville A., Carr Z., Chumak V., Davis S., Demidchik Y. (2006). Cancer consequences of the Chernobyl accident: 20 years on. J. Radiol. Prot..

[17] Veiga L.H.S., Holmberg E., Anderson H., Pottern L., Sadetzki S., Adams M.J., Sakata R., Schneider A.B., Inskip P., Bhatti P. (2016). Thyroid cancer after childhood exposure to external radiation: An updated pooled analysis of 12 studies. Radiat. Res..

[18] Kurohama H., Matsuda K., Kishino M., Yoshino M., Yamaguchi Y., Matsuu-Matsuyama M., Kondo H., Mitsutake N., Kinoshita A., Yoshiura K.-I. (2021). Comprehensive analysis for detecting radiation-specific molecules expressed during radiation-induced rat thyroid carcinogenesis. J. Radiat. Res..

[19] Christov K. (1975). Thyroid cell proliferation in rats and induction of tumors by X-rays. Cancer Res..

[20] Kaspareit-Rittinghausen J., Wiese K., Deerberg F., Nitsche B. (1990). Incidence and morphology of spontaneous thyroid tumours in different strains of rats. J. Comp. Pathol..

[21] Seo J.H., Agarwal E., Bryant K.G., Caino M.C., Kim E.T., Kossenkov A.V., Tang H.-Y., Languino L.R., Gabrilovich D.I., Cohen A.R. (2018). Syntaphilin Ubiquitination Regulates Mitochondrial Dynamics and Tumor Cell Movements. Cancer Res..

[22] Hwang M.J., Bryant K.G., Seo J.H., Liu Q., Humphrey P.A., Melnick M.A.C., Altieri D.C., Robert M.E. (2019). Syntaphilin Is a Novel Biphasic Biomarker of Aggressive Prostate Cancer and a Metastasis Predictor. Am. J. Pathol..

[23] Zhang S.Y., Xu M.J., Wang X. (2018). Adrenomedullin 2/intermedin: A putative drug candidate for treatment of cardiometabolic diseases. Br. J. Pharmacol..

[24] Nagasaki S., Fukui M., Asano S., Ono K., Miki Y., Araki S.i., Isobe M., Nakashima N., Takahashi K., Sasano H. (2014). Induction of adrenomedullin 2/intermedin expression by thyroid stimulating hormone in thyroid. Mol. Cell. Endocrinol..

[25] Kim J.T., Lim M.A., Lee S.E., Kim H.J., Koh H.Y., Lee J.H., Jun S.M., Kim J.M., Kim K.H., Shin H.S. (2022). Adrenomedullin2 stimulates progression of thyroid cancer in mice and humans under nutrient excess conditions. J. Pathol..

[26] Bartucci R., Salvati A., Olinga P., Boersma Y.L. (2019). Vanin 1: Its Physiological Function and Role in Diseases. Int. J. Mol. Sci..

[27] Guan W., Xu J., Shi Y., Wang X., Gu S., Xie L. (2023). VNN1 as a potential biomarker for sepsis diagnosis and its implications in immune infiltration and tumor prognosis. Front. Med..

[28] Traynelis S.F., Wollmuth L.P., McBain C.J., Menniti F.S., Vance K.M., Ogden K.K., Hansen K.B., Yuan H., Myers S.J., Dingledine R. (2010). Glutamate Receptor Ion Channels: Structure, Regulation, and Function. Pharmacol. Rev..

[29] Andersson B., Langen B., Liu P., López M.D. (2023). Development of a machine learning framework for radiation biomarker discovery and absorbed dose prediction. Front. Oncol..

[30] Bademler S., Ucuncu M.Z., Yasasever C.T., Serilmez M., Ertin H., Karanlık H. (2019). Diagnostic and Prognostic Significance of Carboxypeptidase A4 (CPA4) in Breast Cancer. Biomolecules.

[31] Lei X., Liu D., Song D., Fan J., Dai G., Yang L. (2022). Knockdown of carboxypeptidase A4 (CPA4) inhibits gastric cancer cell progression via cell cycle arrest and apoptosis. J. Gastrointest. Oncol..

[32] Choi Y.-S., Jeon M.J., Doolittle W.K.L., Song D.E., Kim K., Kim W.B. (2024). Macrophage-Induced Carboxypeptidase A4 Promotes the Progression of Anaplastic Thyroid Cancer. Thyroid.

[33] Li W.-M., Chan T.-C., Wei Y.-C., Li C.-F., Ke H.-L., Wu W.-J., Hsu C.-C., Wang S.-C., Yeh C.-F. (2023). Downregulation of CRTAC1 in Urothelial Carcinoma Promotes Tumor Aggressiveness and Confers Poor Prognosis. Front. Biosci..

[34] Tan L., Zhang H., Ding Y., Huang Y., Sun D. (2024). CRTAC1 identified as a promising diagnosis and prognostic biomarker in lung adenocarcinoma. Sci. Rep..

[35] Yang J., Fan L., Liao X., Cui G., Hu H. (2021). CRTAC1 (Cartilage acidic protein 1) inhibits cell proliferation, migration, invasion and epithelial-mesenchymal transition (EMT) process in bladder cancer by downregulating Yin Yang 1 (YY1) to inactivate the TGF-beta pathway. Bioengineered.

[36] Matsuu-Matsuyama M., Shichijo K., Matsuda K., Fujimoto N., Kondo H., Miura S., Kurashige T., Nagayama Y., Nakashima M. (2021). Age-dependent effects on radiation-induced carcinogenesis in the rat thyroid. Sci. Rep..

[37] Meadows S.K., Dressman H.K., Muramoto G.G., Himburg H., Salter A., Wei Z., Ginsburg G.S., Chao N.J., Nevins J.R., Chute J.P. (2008). Gene expression signatures of radiation response are specific, durable and accurate in mice and humans. PLoS ONE.

[38] Paul S., Smilenov L.B., Elliston C.D., Amundson S.A. (2015). Radiation Dose-Rate Effects on Gene Expression in a Mouse Biodosimetry Model. Radiat. Res..

[39] Jafer A., Sylvius N., Adewoye A.B., Dubrova Y.E. (2020). The long-term effects of exposure to ionising radiation on gene expression in mice. Mutat. Res. Mol. Mech. Mutagen..

[40] Lee J.-R., Ahn K., Kim Y.-J., Jung Y.-D., Kim H.-S. (2012). Radiation-Induced Human Endogenous Retrovirus (HERV)-R env Gene Expression by Epigenetic Control. Radiat. Res..

[41] Seong K.M., Cenci G. (2022). Editorial: The Genetic and Epigenetic Bases of Cellular Response to Ionizing Radiation. Front. Genet..

[42] Larsson M., Rudqvist N., Spetz J., Shubbar E., Parris T.Z., Langen B., Helou K., Forssell-Aronsson E. (2020). Long-term transcriptomic and proteomic effects in Sprague Dawley rat thyroid and plasma after internal low dose 131I exposure. PLoS ONE.

