# Supplementary material for: Identification of Thyroid Genes Whose Expression Is Altered by Neonatal Irradiation in Rats
Source: Int J Mol Sci. 2025 Feb 21;26(5):1874. doi: 10.3390/ijms26051874 (PMC11899105; doi:10.3390/ijms26051874)
Supplement: Supplementary file 1 [file ijms-26-01874-s001.zip › Tables S1-S3 and Figures S1 and S2.pdf]

Table S1. Serum total T3, total T4, and TSH levels.

Group	Total T3 (ng/ml)	Total T4 (ug/dL)	TSH (ng/ml)
1 week old			
1w0Gy	2.0±0.13	7.5±0.20	3.4±0.24
1w4Gy	1.7±0.16	7.7±0.10	4.2±0.29
4 weeks old			
4w0Gy	1.7±0.07	7.9±0.25	4.0±0.24
4w4Gy	1.8±0.16	7.8±0.11	4.4±0.35
8 weeks old			
8w0Gy	1.8±0.19	7.9±0.16	4.5±0.38
8w4Gy	2.0±0.13	8.5±0.36	4.5±0.49
4 months old			
4m0Gy	1.8±0.09	7.1±0.29	3.9±0.15
4m4Gy	1.7±0.12	7.3±0.37	3.9±0.32

The numbers of rats were 7 rats / group in 1-week-old and 4-month-old groups, and 5 rats / group in 4-week-old and 8-week-old groups.

Table S2. RNA-Seq results.

a. RNA-Seq results - 1w0Gy vs 1w4Gy.

Gene	RNA-Seq		Q-PCR	
	fold	*P*adj	fold change	
up-regulated				
Miip	3.65	0.011	0.95±0.08	Migration/invasion inhib protein
Eno2	3.53	0.009	0.93±0.08	Enolase 2
Cdkn1a	3.08	0.000	2.3±0.14**	Cyclin-dependent kinase inhib 1A
Ass1	2.72	0.013	1.6±0.24	Argininosuccinate synthase 1
Snph	2.43	0.001	1.9±0.24**	Syntaphilin
Adm2	2.37	0.019	1.9±0.23**	Adrenomedullin 2
Vnn1	2.22	0.011	1.8±0.24*	Vanin 1
Gria3	2.03	0.000	1.5±0.06**	Glutamine receptor A3
Per1	1.88	0.019	1.5±0.33	Period circadian regulator 1
B9d1	1.75	0.002	0.83±0.05	B9 domain-containing protein 1
Cpa4	1.69	0.013	1.4±0.09**	Carboxypeptidase A4
Slc4a4	1.58	0.027	1.0±0.08	Solute carrier family 4 member 4
Tfcp2l1	1.54	0.036	1.2±0.09	Transcription factor CP2-like 1
down-regulated				
RT1-DMa	0.30	0.001	0.91±0.07	RT1 class II locus DMa
Srm	0.34	0.000	0.88±0.06	Spermidine synthase
Crtac1	0.39	0.009	0.44±0.03*	Cartilage acidic protein 1
C6	0.45	0.013	0.69±0.13	Complement C6
Nefh	0.46	0.006	0.46±0.09	Neurofilament heavy
Cidea	0.49	0.013	0.38±0.08	Cell death-ind DFFA-like effector A

b. RNA-Seq results - 4m0Gy vs 4m4Gy

Gene	RNA-Seq		Q-PCR	
	fold	*P*adj	fold change	
up-regulated				
Cdkn1a	2.49	0.000	2.3±0.21**	Cyclin-dependent kinase inhib 1A
Ephx1	1.60	0.000	1.4±0.08**	Epoxide hydrolase 1
Pinlyp	1.55	0.033	1.0±0.13	Phospholipase A2 inhib Ly6/Plaur
down-regulated				
Wdr77	0.33	0.033	0.83±0.03*	WD repeat domain 77

*, ** indicate statistical differences from each control (0 Gy) at p<0.05 (*), or p<0.01 (**).

Table S3. Q-PCR primers.

Gene	GenBank Accession#	Q-PCR primer sequences (5' -> 3') forward	reverse
Adm2	NM_201426	CTTGCTACGGTTCATCTGCCT	CGATGGCTGAGATTCTGGACTT
Ass1	NM_013157	GCCTCAAATTCGCAGAGCTC	GGATGTACACCTGGCCCTTG
B9d1	NM_001105786	AGTTCACAAGCTGGTTCATGGG	TCATGTCCTTGGTCACCACATT
C6	NM_176074	ACTGTGTCTGCCTATTGCCCC	TTTCCACCTTCCTGTTTGCAC
Cdkn1a	NM_080782	TGTCCGACCTGTTCCACACA	CGTCTCAGTGGCGAAGTCAA
Cidea	NM_001170467	CTGTCTCAATGTCAAAGCCACC	ATAAACCAGGAACTGTCCGGTC
Cpa4	NM_001109346	AGGAAGCCGCTGTATTGGAG	GCATTTGAAGTTCCCATGCTTT
Crtac1	NM_134401	TTGCATGGACACCAATGAATG	GGAATACCCACCTAGAAAGGCC
Eno2	NM_139325	TTGGATTTCAAGTCTCCCGCT	GAACGTGTCCTCGGTTTCTCC
Ephx1	NM_001034090	TCCCTGGATGATCTGCTGGT	CAGTGGGCACAAAGACCTTCA
Gria3	NM_032990	CAAGGACTCCGGGAGTAAGGA	GGACTCTGCCCGTGATTTGTAA
Miip	NM_001017450	GTTTCCTGAGCCTCTGGATCC	TACTGGTGTGGAGGGTCCATG
Nefh	NM_012607	AAAGCCAAAGAACCCCCAAA	TTCTGTGAGAGGCTCCTTCTCC
Per1	NM_001034125	GGAACTGGGTGCTGTGCACT	TGGCCAGGATCTTGAACACTG
Pinlyp	NM_001107488	ATCAACACGAAATTTGCCACG	TCTTCACTCTCCCCTGCCAG
RT1-DMa	KC222886	ACCTGGCAGCATCATTTCGT	GTGCGTCACAGTGCAGGAGTA
Slc4a4	NM_053424	TCATGGCTCTTCCCTGGTATGT	GGGTTCCAGTGACTCGTTGTTC
Snph	NM_001106525	TGCAGTACCAGCCTGACCTG	TCCGGCTCGTCACCTACTGT
Srm	NM_053464	AGCCCTCAAAGAAGATGGCAT	CGATCTGGCCACTGGGATAG
Tfcp2l1	NM_001107170	ACCTGCTCCCATCAGCCTCT	CCACAGACCTGGACCAAATCAT
Vnn1	NM_001025623	TTTGGAACGCGGTATGTCTTC	CCTCCCAAAGAGACCGACTG
Wdr77	NM_001008771	TTGTGGACACCAAGAATGCAA	GCTTGAATCCAGCACAGCAA

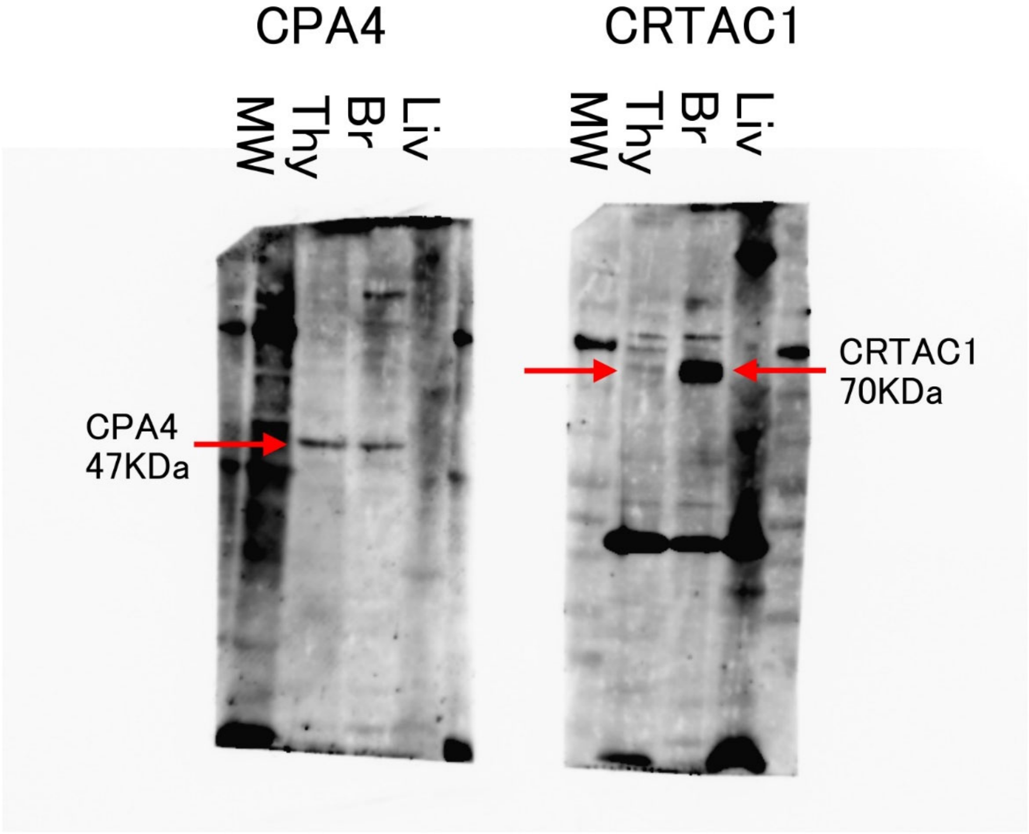

Figure S1. Western blot validation of Antibodies to CPA4 and CRACT1.

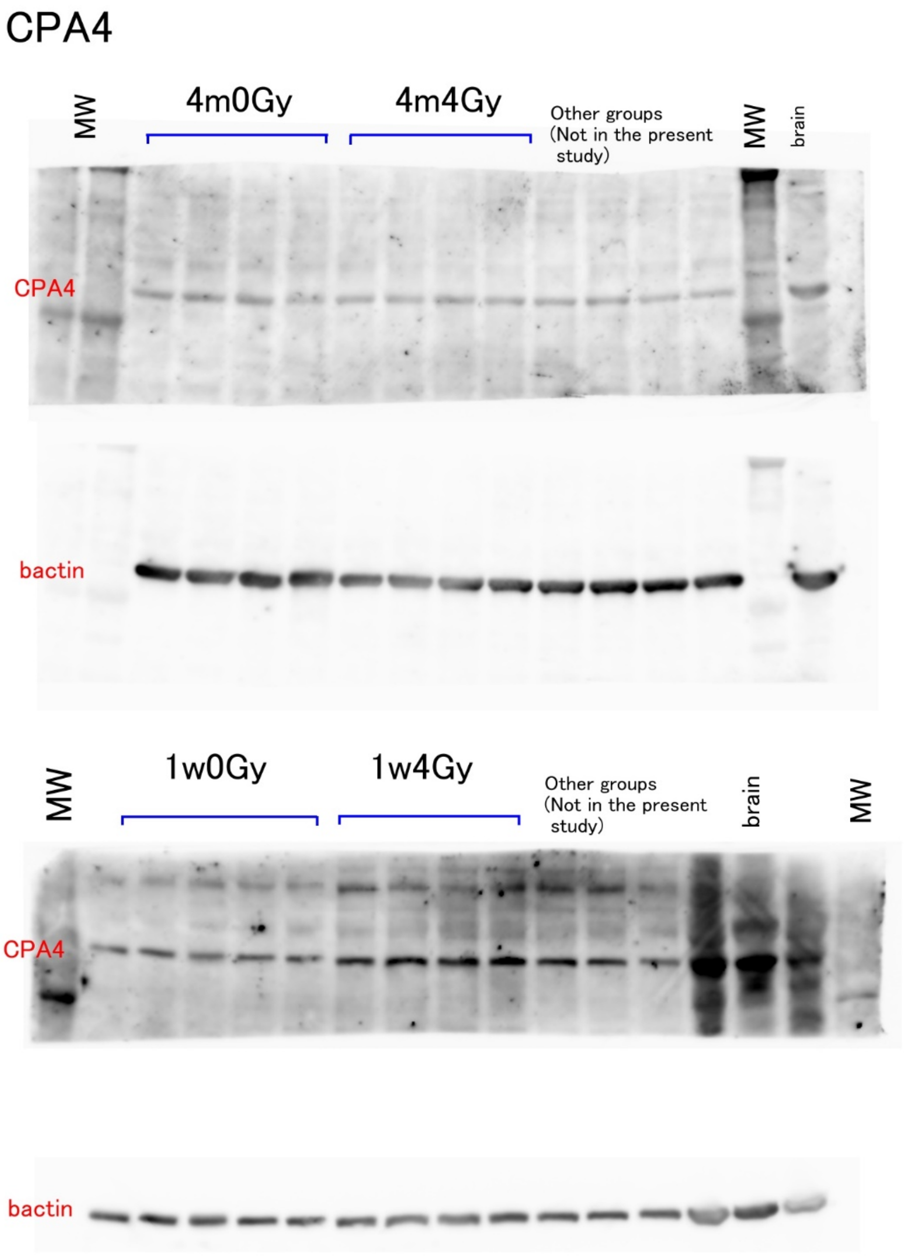
CPA4
MW
4m0Gy
4m4Gy
Other groups
(Not in the present study)
MW
brain
CPA4
bactin
MW
1w0Gy
1w4Gy
Other groups
(Not in the present study)
brain
MW
CPA4
bactin

(a)

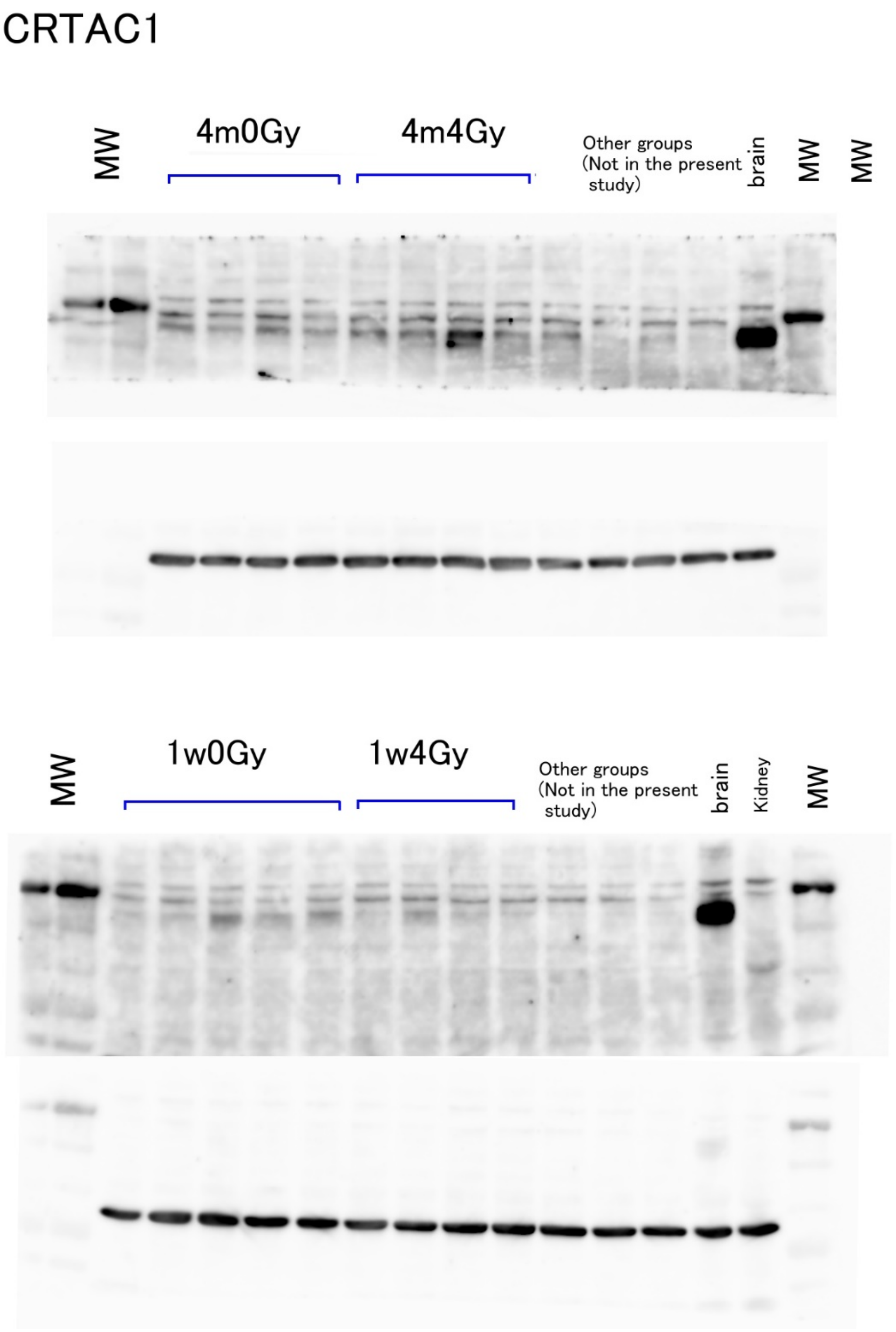

(**b**)

Figure S2. (**a**) CPA4 pro-tein expressions in the thyroid. (**b**) CRTAC1 protein expressions in the thyroid.
